# Does the timing of ACL reconstruction affects meniscal tear type?: A retrospective cohort study

**DOI:** 10.1097/MD.0000000000047796

**Published:** 2026-02-28

**Authors:** Mehmet Ali Sabir, Riza Berker Özbek, Selahaddin Aydemir

**Affiliations:** a Department of Orthopaedics and Traumatology, Kastamonu Education and Research Hospital, Kastamonu, Turkey.

**Keywords:** ACL reconstruction, bucket-handle tear, meniscal tears, meniscus repairability, surgical delay

## Abstract

Anterior cruciate ligament (ACL) injuries are often accompanied by meniscal tears, and the timing of reconstruction may influence tear characteristics. The aim of this study was to evaluate the effect of the timing of ACL reconstruction (ACLR) on meniscal injuries and to examine the association of surgical delay with the incidence, type and repairability of meniscal tears. This retrospective study included patients who underwent ACLR between January 2023 and January 2024. Demographic data, mechanism of injury, time between injury and surgery, and arthroscopic video recordings were obtained from the hospital system. The type of meniscal tears (longitudinal, horizontal, radial, bucket handle, ramp lesion, root tear, complex), tear localization (anterior, middle, posterior) and zonal classification were recorded according to the Cooper system. Statistical analyses were performed to evaluate the incidence of meniscal tears, changes related to surgical timing and repairability rates. A total of 46 patients were included in the study. The mean age of the patients was 30.8 years (17–52) and the mean interval between injury and surgery was 9.4 months (6–18). The meniscal tear rate was 63% and 66% of these tears were observed in the medial meniscus. Bucket-handle tears were the most common tear type and were significantly more common in the medial meniscus (*P* <.001). Despite this, all meniscal tears were found to be repairable. Our study shows that delayed ACLR increases the frequency of medial meniscal and bucket-handle tears. However, repairability of meniscal tears was preserved despite surgical delay. This finding suggests that surgical timing alone may not be the sole determinant and that tear morphology, surgical technique and patient factors may also play an important role. Further large scale, prospective and randomized controlled studies are needed to determine the optimal timing of ACLR for meniscal preservation.

## 1. Introduction

In the anteroposterior stability of the knee joint, the anterior cruciate ligament (ACL) functions as the primary stabilizer and the medial meniscus (MM) functions as the secondary stabilizer. When there is a deterioration in ACL function, the MM provides primary stabilization in the anterior posterior stabilization of the knee.^[1]^ ACL injuries have significant negative effects on quality of life and may lead to the development of knee osteoarthritis in the long term.^[2]^ ACL injuries are common, especially among athletes and physically active populations. Age, gender, body mass index and physical activity level are effective in ACL injury.^[3]^ It is more likely to be seen in adolescents and women.^[4]^ The ACL injury is generally seen after noncontact (70%) and contact (30%).^[5]^ The mechanism of the injury is often sudden and excessive rotation of the knee while the lower extremity is fixed on the ground.^[6]^ There are studies in the literature on the coexistence of ACL injuries and meniscus injuries. Meniscus injuries may be accompanied in 44.4 to 63% of cases.^[1,7]^ Accompanying meniscus lesions may vary in acute and chronic ACL injuries. Injuries to the lateral meniscus (LM) may be more common in acute ACL injuries, and injuries to the MM is more common in chronic ACL injuries.^[1,8]^ Failure to properly treat meniscal injuries may lead to graft failure after ACL reconstruction.^[9]^ Although meniscus injuries accompanying chronic ACL injuries have been described in the literature, there are limited studies on the type, location and zone of these injuries. The aim of this study is to increase awareness about meniscus injuries accompanying chronic ACL injuries by making a detailed analysis of meniscus injuries accompanying chronic ACL injuries.

## 2. Methods

Patients who underwent surgery at Kastamonu Education and Research Hospital, a tertiary medical facility, for ACL injury between January 2023 and January 2024 were included in our study. The study was approved by the Kastamonu University Ethics Committee (approval no: 2024-KAEK-61). Age, operated extremity, gender, injury mechanism, injury to surgery time, arthroscopy video recording data of the patients were taken from the hospital system. Data on the affected meniscus, type of injury (longitudinal, horizontal, radial, bucket handle, ramp lesion, root, complex), tear site (anterior, middle, posterior) and meniscus injury were recorded with the Cooper classification.^[10]^ According to the Cooper classification, each meniscus is named as 1,2,3 respectively, from the periphery to the medial in the axial plane.

The mechanism of injury is defined in 2 groups: Contact and noncontact.

Inclusion criteria: Having ACL reconstruction (ACLR) surgery; Be over 18 years old. However, 1 17-year-old patient with complete skeletal maturity and with the consent of guardians was also included.

Exclusion criteria: Having additional ligament injury other than the ACL;

Patients without arthroscopy video recording; Lack of demographic data; History of previous knee surgery (history of fracture or arthroscopic surgery); Acute injuries (<6 weeks); Avulsion-type injuries; Kellgren-Lawrence grade ≥ 2 osteoarthritis.

**Figure 1.**
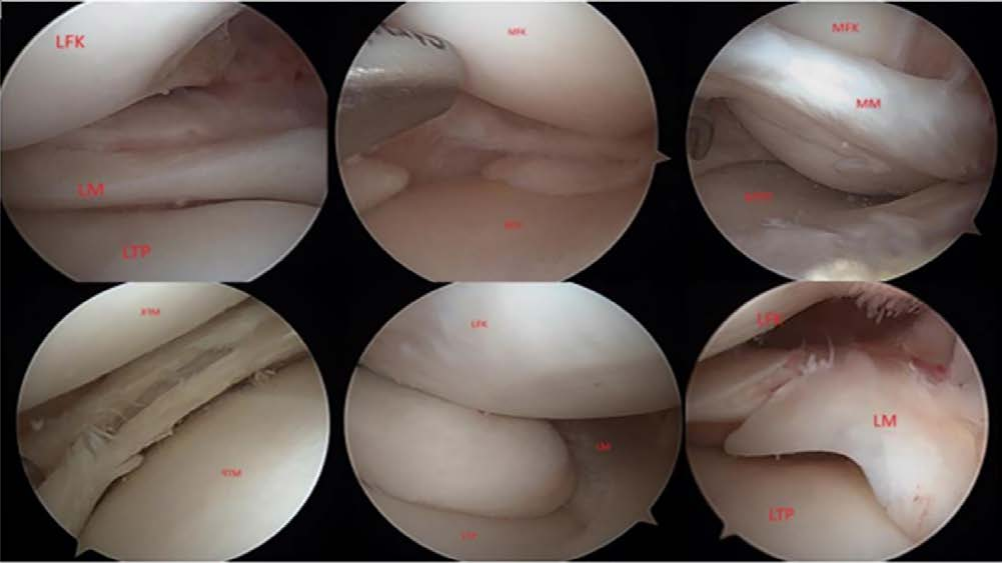
(A–F): Type of meniscal injuries found in different patients.

A total of 46 patients were included in the study after the inclusion and exclusion criteria were applied. The patients included in the study were operated on by a single surgeon (MAS). Anterolateral, medial and far medial portals were used during the surgery. Medial and LM examinations were performed before ACL reconstruction. Posterior compartment imaging was performed with the modified Gillquist view as standard.^[11]^ Interventions were performed in patients with meniscus injuries according to the type and size of the tear (Fig. 1A–F). After meniscus treatment, ACL reconstruction was performed with hamstring graft.

Statistical analysis was performed using SPSS v25.0 (IBM Corp., Armonk). The chi-squared goodness of fit test was utilized to compare meniscal injury types. Student *t* test were used to compare clinical and demographical characteristics of 2 groups. *P*-values of <.05 were considered significant.

## 3. Results

Total of seventy-two patients were operated for ACL rupture between January 2023-January 2024. Sixteen patients’ injury time was below 6 weeks. 6 patients’ video record was missing, 2 patients had concomitant ligament injury and 2 patients had previous knee surgery. A total of 46 patients were eligible for final analysis after exclusion criterion were applied. Table 1 reveals the sociodemographic data of the patients. The mean age of the patients were 30.8 years (range 17–52) (Table 1). The mean time from injury to operation was 9.4 (6–18) months. The most of the patients were male (n = 38) and also male gender was predominant among patients with meniscal injuries (n = 24). The age and sex of the patients were not associated with the presence of meniscal injury (*P* = .238) and also with the meniscal injury types (*P* = .763) and localizations (*P* = .187). We found no difference between 29 patients had concomitant meniscal injuries. All meniscal repairs were done concomitantly with ACL reconstruction. The other 17 patients had intact menisci. Among patients with meniscal injuries 19 patients (66%) had medial sided meniscal injury and 10 patients (34%) had lateral sided meniscal injury. Medial meniscal injuries were significantly more common than lateral meniscal injuries (*P* <.001). There were no perioperative complications including infection and neurovascular injuries. Table 2 summarizes the meniscal injury locations. The posterior horn was injured in most of the meniscal injuries (86%). The mechanism of injury was predominantly noncontact injuries (n = 38). There was no difference between mechanism of injury and concomitant meniscal injury (*P* = .4). We also found no difference between mechanism of injury and related meniscal injury type (*P* = .767). The Cooper zones of the meniscal injuries and the vascular zones of the menisci did not differ in terms of mechanism of injury, age, sex and side of the menisci. We detected no difference in meniscal injury types in patient group, but bucket-handle tears were significantly more common in MM injuries (*P* <.001) (odds’ ratio: 35). Also, even if we could not manage to detect a statistically significance, there was a trend towards horizontal meniscal injuries were more common in lateral menisci.

**Table 1:** Demographics of the patients.

	Mean (range)
Age at surgery	30.8 (17–52)
Gender (M: F)	38:8
Right: left	26:20
Medial: lateral	19:10

**Table 2:** Meniscal injury localizations.

Tear location	Horizontal	Bucket-handle	Radial	Ramp	Complex	Lateral root tear	Longitudinal	Sum
Posterior horn	2	–	1	4	2	2	2	13
Body	1	–	–	–	–	–	2	3
Posterior horn and body	1	10	–	–	1	–	–	12
Total meniscus	–	1	–	–	–	–	–	1
Sum	4	11	1	4	3	2	4	29

## 4. Discussion

Meniscal lesions accompanying ACL injuries have been studied by many researchers in the literature, and the prevalence of meniscal tears during ACL reconstruction (ACLR) has been reported to vary between 24% and 79%.^[1,12–15]^

This rate was found to be 63% in our study and was reported to be 21.8% in a study conducted on a Turkish database.^[9]^ However, the fact that this study was based on operation coding in the national database data is an important limitation. In our study, the rates obtained by analyzing the direct records obtained during arthroscopy are thought to be more realistic.

In our study, MM tears were found to be more common in chronic ACL injuries (66%) and these findings were consistent with the existing data in the literature.^[1,16–19]^ Keyhani et al reported that delaying ACLR for more than 3 months increased the incidence of meniscal injuries, especially medial meniscal injuries and ramp lesions.^[1]^ In a systematic review and meta-analysis by Prodromidis et al, it was found that delaying ACLR for more than 3 months significantly increased the risk of MM injury.^[19]^ Ding et al emphasized that delaying ACLR for more than 9 months was associated with more meniscus and cartilage injuries.^[16]^ Similarly, Cristiani et al reported that the risk of MM injury increased significantly when ACLR was delayed for more than 12 months.^[20]^ In line with these findings, it is recommended that ACLR be performed within 12 months to prevent MM tears following ACL injury. It is known that the MM is more vulnerable because the knee becomes unstable after ACL tear and the risk of reinjury of the MM increases as a result of the “second blow” phenomenon.^[18]^

Ghodadra et al emphasized that the types of meniscal tears may change with the delay of ACLR.^[21]^ Kawashima et al showed that delaying ACLR for more than 12 months increased the incidence of medial meniscal bucket-handle tears.^[22]^ In their study, it was found that medial meniscal bucket-handle tears were significantly more frequent in the acute group when ACLR was delayed for more than 12 months compared to the acute group (33.3% vs 3%).^[22]^ Hagino et al found that bucket-handle tears occurred more frequently if ACLR was delayed for more than 8 weeks.^[13]^ Guenther et al analyzed 112 adolescents with ACL tear and reported that the frequency of medial meniscal tears, especially bucket-handle tears, gradually increased 1 year or more after ACL injury.^[23]^ It reveals that the timing of ACLR is a critical factor in terms of meniscal preservation. However, since MR images of the patients at the time of injury were not available in previous studies, it should be considered that the trauma that occurred during ACL injury may have developed together with meniscal tear. This situation creates uncertainty about the reasons for the higher incidence of medial meniscal lesions in chronic ACL reconstruction surgeries. Therefore, further studies including patients with MR imaging in the early postinjury period are warranted. On the other hand, although the necessity of surgical intervention after ACL injury has been emphasized in the literature, the decision-making process of patients for surgery may be prolonged for different reasons. The occurrence of mechanical symptoms, especially knee locking and pain, may lead patients to readmission. This may explain the high rate of bucket-handle tears found in our study. In the literature, there are not enough studies on why patients delay their operations. However, considering that bucket-handle tears more frequently cause mechanical symptoms, it is possible that these patients may have sought treatment because of more prominent complaints.

In our study, it was observed that the time between injury and surgery had no significant effect on the repairability of meniscal tears. Everhart et al reported that delaying ACLR for more than 8 weeks was associated with irreparable medial meniscal tears.^[24]^ Similarly, Tomihara et al reported that a delay of more than 3 months and Chhadia et al reported that a delay of more than 12 months decreased MM repair rates.^[25]^ In our study, all meniscal tears were repairable despite a mean time to surgery of 9 months. The repairability of meniscal tears is related not only to the duration and morphology of the injury, but also to various factors such as patient expectations, surgical technique, rehabilitation process and preoperative joint stability. Therefore, prospective studies with large patient groups are needed to better understand the effect of surgical timing on meniscal repair success.

The effect of age on meniscal injuries is also a controversial issue. Cristiani et al reported an increased risk of both MM and LM tears in patients aged 30 years and older.^[20]^ However, Tomihara et al did not find a significant relationship between age and MM or LM tears.^[26]^ Although we could not directly compare meniscal injuries according to age in our study due to limited sample size, medial meniscal tears were found to be more common in our patient group with a mean age of 30 years. This finding is consistent with the data in the literature that medial meniscal tears are more common. However, further studies in larger sample groups are necessary to better understand the effect of age on meniscal injuries and to determine the accuracy of this relationship.

In our study, it was observed that the Cooper system has certain limitations in the classification of meniscal tears. In particular, there is no clear standard in the literature regarding the exact location of bucket handle, radial and horizontal tears. In the literature, it has been reported that the Cooper classification is insufficient in defining the tear region in detail and there are inconsistencies in the classification of these regions in different studies.^[1,27]^ The ISAKOS classification provides a more detailed evaluation of meniscal tears and includes criteria such as tear depth, circumferential width, radial localization, tear pattern and tissue quality, but it has been reported that this system also has some limitations in terms of interobserver reliability.^[28]^ Therefore, in our study, instead of using the Cooper classification, we classified meniscal tears into posterior, middle and anterior zones. In addition, we considered tears involving more than 1 zone as a separate group. Similarly, it should be taken into consideration that meniscal tears may affect not only the localization but also more than 1 zone in the red-red, red-white and white-white zones. In the current literature, although the zonal location and vascularization status of the tear are evaluated in studies on the repairability of meniscal tears, there are not enough methodological standards on how these zones are classified.^[28]^ Another limitation in these studies may be the inadequate identification of meniscal tears by magnetic resonance imaging before surgery.^[28]^ In previous studies, it was reported that the intraoperative confirmation rates of meniscal tears identified by magnetic resonance imaging were variable.^[1]^ This suggests that different diagnostic approaches may be required especially in the evaluation of bucket handle and complex tears. In future studies, the development of a more comprehensive and standardized system for the classification of meniscal tears will contribute to the improvement of diagnostic accuracy and treatment decisions.

Our study has some limitations. Firstly, the sample size is limited and this may limit the generalizability of the results. Secondly, the reasons for surgical delays could not be clearly determined for each patient, and these delays may be largely due to referral processes from other clinics to our hospital. In addition, the dates of injury used to calculate surgical delays were self-reported by the patients, which may have introduced errors in timing. Third, selection bias may have occurred because patients were not randomly assigned to different surgical delay groups. In particular, patients presenting late to surgery may have a higher risk of joint degeneration, which may affect the nature of meniscal tears and repairability rates. In the literature, it has been emphasized that it is difficult to determine the exact effect of timing of surgery on meniscal preservation due to the lack of randomized controlled studies.^[29]^ Considering these limitations, prospective and randomized controlled studies involving larger patient populations are needed to better understand the effect of surgical timing on meniscal tears.

## 5. Conclusion

This study highlights the impact of the timing of ACLR on meniscal injuries. Our findings show that delayed ACLR is associated with an increase in medial meniscal tears and bucket-handle tears in particular. However, despite an average surgical delay of 9.4 months, all meniscal tears were found to be repairable. This suggests that not only timing but also tear morphology, patient expectations, and surgical techniques affect repair success. The high rate of bucket-handle tears in our study may be explained by the fact that these tears cause mechanical symptoms and refer patients to surgery. In the current literature, there is no clear consensus on the optimal surgical timing for meniscal preservation. Therefore, prospective and randomized studies with larger patient groups are needed. Furthermore, more reliable classification systems need to be developed to improve consistency in the diagnosis and treatment of meniscal tears.

## Author contributions

**Conceptualization:** Mehmet Ali Sabir.

**Data curation:** Mehmet Ali Sabir, Riza Berker Özbek.

**Formal analysis:** Riza Berker Özbek.

**Methodology:** Mehmet Ali Sabir, Riza Berker Özbek.

**Writing – original draft:** Mehmet Ali Sabir, Riza Berker Özbek, Selahaddin Aydemir.

**Writing – review & editing:** Mehmet Ali Sabir, Riza Berker Özbek, Selahaddin Aydemir.
